# Adverse Drug Reactions, Power, Harm Reduction, Regulation and the ADRe Profiles

**DOI:** 10.3390/pharmacy6030102

**Published:** 2018-09-18

**Authors:** Sue Jordan, Patricia A. Logan, Gerwyn Panes, Mojtaba Vaismoradi, David Hughes

**Affiliations:** 1 College of Human and Health Sciences, Swansea University, Swansea SA2 8PP, UK; g.panes@swansea.ac.uk (G.P.); d.hughes@swansea.ac.uk (D.H.); 2 Faculty of Science, Charles Sturt University, Bathurst Campus, NSW 2795, Australia; plogan@csu.edu.au; 3 Faculty of Nursing and Health Sciences, Nord University, 8049 Bodø, Norway; mojtaba.vaismoradi@nord.no

**Keywords:** adverse drug reactions, patient safety, nursing, medicine management, long-term care

## Abstract

The power and influence of healthcare systems comes largely from the ability to prescribe efficacious medicine. However, medicine can sometimes cause harm rather than bring benefits. Systematically checking patients for the adverse effects of medicines, as listed in manufacturers’ literature, would protect patients from iatrogenic harm, but this is rarely undertaken. We argue for the benefits of this approach using the example of the prescription of antipsychotics to older adults. Prescribing antipsychotics to control challenging behaviours associated with dementia is a controversial matter, and regulatory intervention is under discussion. Improved regulatory systems could protect against iatrogenic harm, such as over-sedation, falls, tremor, or drug-induced Parkinsonism. However, measuring the impact and outcomes of regulatory interventions has proved difficult, not least because there are rarely systematic records of all adverse effects of medicines. We indicate how regulatory initiatives to reduce antipsychotic prescribing can be supported by systematic monitoring and documentation of patients’ signs and symptoms of putative adverse drug reactions. Monitoring documentation then provides the rationale and support for professionals’ responses to identified problems. Longitudinal monitoring records would improve understanding of the impact and outcomes of adverse drug reactions (ADRs) on health and wellbeing, and the many costs of ADRs.

## 1. Introduction 

Improvements in population health in Western countries in the first half of the 20th century were the culmination of several factors, including improved nutrition, public health measures, and lifestyle changes [1], but gains since the 1940s are at least partly attributable to better medicine [2,3]. Antimicrobial agents, starting with the sulphonamides, vaccination programmes, and cardiovascular medicines, all contributed to significant reductions in mortality and morbidity [4,5,6]. Pharmaceutical advances transformed treatments in the mental health field, with antipsychotics bringing significant benefits for illness management [7,8,9]. Unfortunately, these benefits have been offset, to an uncertain degree, by new forms of iatrogenic disease associated with the adverse effects of prescribed medicines [10,11]. Antipsychotics have not been exempt from adverse drug reactions (ADRs), as considered in the present paper [12,13]. We reflect on the social context of prescribing, the problem of preventable ADRs, our solution, and its congruence with the logic of regulatory interventions.

## 2. Power via Prescribing

The development of effective medicines did much to legitimise the cultural authority of the medical profession, which exercised a professional monopoly over the prescribing of a wide range of pharmaceuticals. Reflecting on the advances of the 1950s, Dr. Julian Tudor Hart explained that: ‘Doctors who were previously seen as people who could only write a prescription for some cough medicine suddenly wrote a prescription for something that actually saved your life. The act of writing the prescription wasn’t *[SIC]* any more skilled in the second instance than in the first, but the effect was quite different. So doctors took a huge leap forward to becoming much, much more credible than parsons and priests as emissaries of God *[SIC]*’ [14]. Even in an age of ‘patient-centred medicine’, medical professionals remain socially distant from certain sections of the population, including people with mental illness and older patients in long-term care institutions, who are the subject of this paper. Past studies have found that medical care in nursing homes is suboptimal, with poor use of psychotropic medication and shortcomings in pain management [15,16,17]. The near medical monopoly over prescribing means that other members of the care team are rarely as attentive to the side effects of mental health medicines as would be desirable. There is a risk that patients in care homes are disadvantaged by their ‘sick role’ status and unintentional ‘othering’ [18], and risk being labelled by their diagnoses and prescriptions, without adequate oversight of ongoing pharmacotherapeutic regimens. Ways need to be found to increase the effectiveness of medicines’ monitoring by front-line care staff. 

Healthcare professionals routinely underestimate harm and overestimate benefits of prescription medicines [19]. Prescribed medicines benefit between 4% and 25% of patients [20], whilst ADRs affect 7.8% of primary care patients [21], 4.8–37% of people with cognitive impairment [22], and 11.0% of hospitalised patients [23], killing 0.25% of hospitalised patients [24]. Lower figures (1.85%) are reported in Norway, excluding events of unknown aetiology [25]. The inability to distinguish drug-induced signs and symptoms from definitive medical diagnoses tends to result in further prescriptions, increasing the risk of drug–drug and drug–disease interactions [26]. However, systematic processes to check patients for the potential adverse effects of their medicines are rarely used [27,28] and consensus for usage of available tools remains remote [29]. 

## 3. The Adverse Drug Reaction Problem

Preventable ADRs have proved an intractable problem over the last decade, causing 5–8% of unplanned UK hospital admissions [30,31], rising to ~10% amongst older adults [32], and costing the NHS £1 bn–£2.5 bn each year [33]. ADRs assessed as due to avoidable errors are responsible for 712–22,303 UK deaths, costing £98.5 m–£1.6 bn each year [34]. In Australia, 15% of admissions of older people (aged 72–88 years) are due to ADRs, 92% of which are preventable [35], and admissions related to all types of ADRs are associated with 28–32% longer hospital stays [36]. The problem is at least as extensive in developing countries, at ~10% of admissions [37]. However, ~60% of adverse drug events are not recognised and reported as such, which suggests that these figures may be an underestimate [38], and higher figures (11%, 18% and 19.6% in the elderly), are quoted [21,39,40,41]. Most ADRs, adverse drug events (ADEs), and medicine mismanagement (including errors by patients and professionals) are preventable [21,30,31], particularly with additional enhanced monitoring [30,31,42,43,44,45,46,47], but comprehensive systematic multiprofessional approaches are needed to address the problem [27,28] (Table S1).

## 4. Antipsychotics, Challenging Behaviour and Older Adults in Care Homes

Some 50% of residents in UK care homes are prescribed mental-health medicines [48]. Doses in care homes [49] and primary care [16] are often excessive, and the proportion of care home residents exposed to inappropriate medications ranges from 34% [50] to > 50% [51]. ADRs as a result of mental-health medicines can be life-threatening (e.g., cardiac arrhythmias, cardiac hypofunction), or debilitating (e.g., drug-induced Parkinsonism, ataxia, postural hypotension), or subtle, and mistaken for signs of ageing or underlying pathology. They can be overlooked, leading to behaviour problems, xerostomia, constipation, poor food and/ or fluid intake, tremor, restlessness, sedation, pain, double incontinence, or other problems, all causing potential loss of comfort and dignity [27,52]. 

The excess dose-related mortality and incidence of strokes associated with antipsychotics [53] have long been concerns. In a trial of withdrawal of antipsychotics, 25/83 (30%) patients taking antipsychotics and 48/82 (59%) taking placebos survived 36 months [54]. Between 1 in 8 and 1 in 29 people with dementia prescribed antipsychotics suffered a stroke [55]. Drug-induced Parkinsonism occurred in 2–12% patients, and sedation in 15–24% [56]. A consensus is emerging around overprescribing [16,50,51,57,58,59,60]. However, there is no consensus regarding the changes needed to routine care [61,62,63,64,65], and reviewers indicate that evidence for single-profession interventions is equivocal [66], of low quality [21,67], or uncertain [66,68,69,70,71]. The UK Department of Health’s National Dementia Strategy [16], launched in 2009, and the Medicines and Healthcare products Regulatory Agency (MHRA) recommendations [72], have not reduced antipsychotic prescribing in care homes [73], and prescribing of atypical antipsychotics has increased across primary care [74], warranting a closer review of regulatory interventions. Evidence collated for the recent Welsh Government (WG) report [62] showed widespread concern about the continuing overuse of antipsychotics (Box 1).

Box 1 **Use of Antipsychotics in Care Homes: A Report from the National Assembly for Wales.**

During 2017 and 2018, the Health, Social Care and Sport Committee of the Welsh Government, chaired by Dr D. Lloyd, collected evidence and made 11 recommendations to ‘protect some of our most vulnerable citizens’. The report concludes: **Antipsychotic medicines are being prescribed inappropriately, in many cases as a first option rather than a last resort, to treat the behavioural and psychologic symptoms of dementia, often without adequate reviews or records being kept p.61. Testimonies included: **

“… where you have somebody with behavioural or psychological problems relating to their dementia, to routinely prescribe an antipsychotic medication without looking at what is underpinning those behaviours and causing them is wrong, and it does seem to be the default position, which needs to be addressed.” Alzheimer’s Society Cymru, p. 17.

“It is quite simply unacceptable that antipsychotic medication is still being used as a primary response to ‘challenging’ behaviour across many residential care services.” Older People’s commissioner, Wales, p. 17.

“I’d like to see the circumstance whereby people are not caught in this dreadful repeat prescription mechanism, whereby it rolls on and on and on, and you could go for two years and nobody refers to it. Some GPs, some practices, are better at reviewing than others. But, theoretically, because you’re caught up in this repeat prescription mechanism, it could carry on and on and on. I’d like to see—if you’re prescribed an antipsychotic and you have a diagnosis of dementia, there should be a three-month mandatory review.” Care Forum Wales, p. 29.

### What Should Be Done Differently?

Identification of medicine-related incidents as the most prevalent source of unsafe primary care in Wales and England underlines the urgency of the problem [75]. Most ADRs, adverse drug events (ADEs), or side-effects are due to poor monitoring, not poor prescribing [30,31,42,43,44,45,46], and are dose-related [47].

The risk of iatrogenic harm could be addressed as an integral part of individual patient care. Patients need to be systematically monitored or checked for the signs and symptoms of ‘undesirable’ effects as listed in manufacturers’ literature. To this end, we have developed a nurse-led approach to medicines’ monitoring (Box 2). This multidisciplinary intervention is important to complement regulatory initiatives in this area. Searches and reviews indicate there is no alternative comprehensive, systematic patient assessment of problems potentially related to prescribed medicines [28,42,76,77]. We are unaware of a similar outcome-monitoring approach adopted in Norway or Australia [78]; a pharmacist-led de-prescribing education initiative in Australian care homes reduced antipsychotic prescriptions, but not adverse outcomes [79].

Box 2 **The Adverse Drug Reaction (ADRe) Profile.**

**Figure 1:**
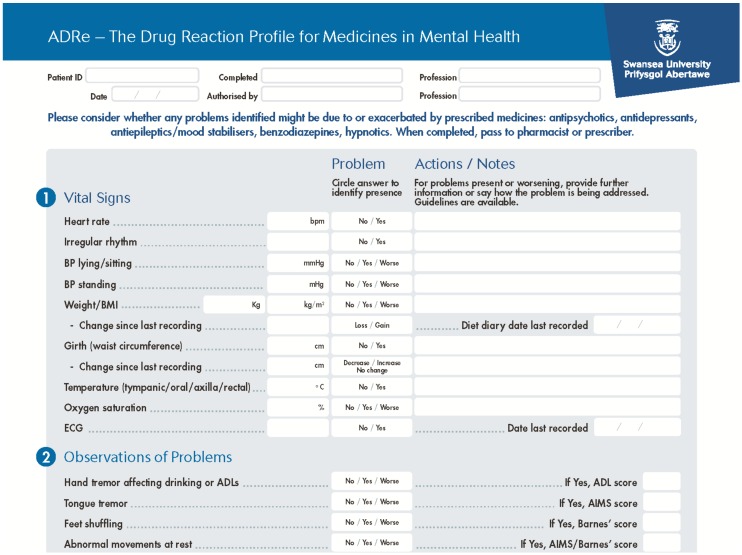
An illustration of the Adverse Drug Reaction (ADRe) Profile for mental health medicines. These are the first questions on the ADRe Profile. This is accompanied by a ‘How to Use’ sheet, and supporting information (Figure 2). To request a copy of ADRe, visit: http://www.swansea.ac.uk/adre/.

**Figure 2:**
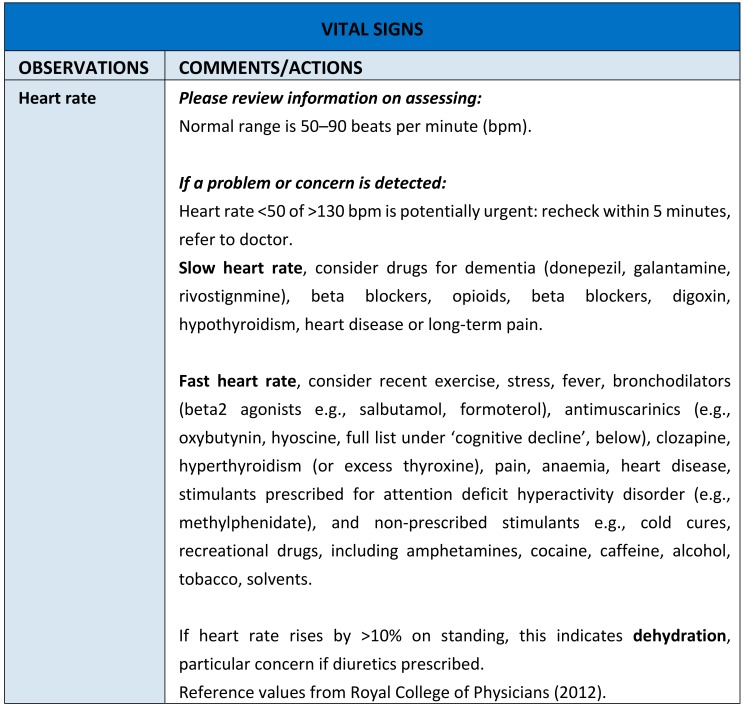
An example of ADRe’s Supporting Information. If a problem is identified, nurses are asked to review the supporting information for that problem, and consider the actions suggested. The suggested aetiologies should be discussed within the multidisciplinary team.

Our intervention, the Adverse Drug Reaction (ADRe) Profile asks nurses to systematically check patients for the manifestation of itemised adverse side effects or undesirable effects of their mental health medicines, as listed in the British National Formulary and manufacturers’ Summaries of Product Characteristics (SmPCs), action problems identified, and share the Profile with pharmacists and/or prescribers. This is a formalised and standardised approach to monitoring all patients prescribed mental health medicines, regardless of diagnostic categories. The ADRe Profile comprises: vital signs, and changes in vital signs, observations, focussed questions or reports of possible ADRs, prevention /health promotion areas likely to be affected by medicines, including diet, medicine use, service user’s perspectives, and care plan modifications (http://www.swansea.ac.uk/adre/requestforadretool/) (Figure 1). Nurses pass the completed profile with problems highlighted to pharmacists and prescribers to inform medication review. The supporting information suggests which medicines or conditions might be causing the indicated problems and what further information might be of assistance (Figure 2). The list of problems is then juxtaposed with the medicines’ record, and the multidisciplinary team determine possible aetiologies of problems identified, and whether medicines need to be changed.

The ADRe Profile for mental health medicines was introduced to address nurses’ and patients’ concerns that ADRs were neither recognised nor communicated to prescribers. The benefit of listing problems in one document, which can be passed to prescribers reviewing medication, is that these problems then get addressed, doses of antipsychotics and sedatives are reduced, and patients’ signs and symptoms resolve. Our trials provide evidence that ADRe works [27,80], supported by other studies [81,82,83,84] (Table 1). ADRe and its supporting information empower nurses by a democratisation and transfer of useful, necessary, and practical knowledge of pharmacotherapeutics and medicine to nurses [85,86,87]; it was developed in Wales, a country with egalitarian traditions [88].

**Limitations of the ADRe Studies**

*Generalisation*

ADRe was developed in post-industrial South West Wales. Findings from a small European country, where most of the population are in an EU convergence zone (GDP < 75% of the EU mean), cannot necessarily be extrapolated to different populations. Without further studies to confirm transferability, generalisation of findings depends on logical inferences. However, medicine mismanagement is an international problem, in urgent need of effective interventions [89].

*Volunteer Bias*

We acknowledge the potential for volunteer bias in all research designs. Only policy initiatives can determine whether our findings will transfer to struggling organisations, as they are unlikely to volunteer for research projects, and may be less likely to have sufficient staff to offer support to those withdrawing from medicines. Participant recruitment to our studies was at the discretion of nurses, and we cannot discount the possibility of selection bias.

*Nurses’ Reporting*

ADRe relies on nurses’ reports; some care home residents are non-verbal and some mental health service users lack communication skills [84]. ADRe is vulnerable to nurses’ interpretations, and we acknowledge that nurses may under-report patients’ ADRs [76]. Although ~50% of ADRe items can be identified in care homes’ notes, the information takes ~1 h to retrieve [27], and prescribers are limited to 7–15 min per patient. Therefore, ADRe increases the information passed to prescribers.

*Time Needed to Complete ADRe*

Initially, ADRe asks nurses or their assistants to do more work by spending 10–30 min documenting problems with each patient, and acting on problems identified; we estimate this costs £20 [27], which can only be compensated by reduction in patients’ demands following improved patient welfare. This initial barrier to uptake usually recedes with familiarity and regular use. If problems are addressed, patients benefit, medicines are optimised, and patients are calmer (Table 1); these clinical improvements persuade nurses to continue with ADRe. The diversion of resources to ADR management may be cost-effective [77], but further work is needed to explore any unintended consequences, such as neglect of other documentation.

*Evaluating the Outcomes of ADRe*

ADRe is comprehensive, with ~80 items, most of which are clinically important. Therefore, rather than defining a single ‘outcome measure’, we have used a composite ‘number or problems identified and addressed’, and described clinical gains. Table 1 has illustrative examples.

## 5. Regulatory Interventions to Modify Prescribing and Outcomes

**Figure 3:**
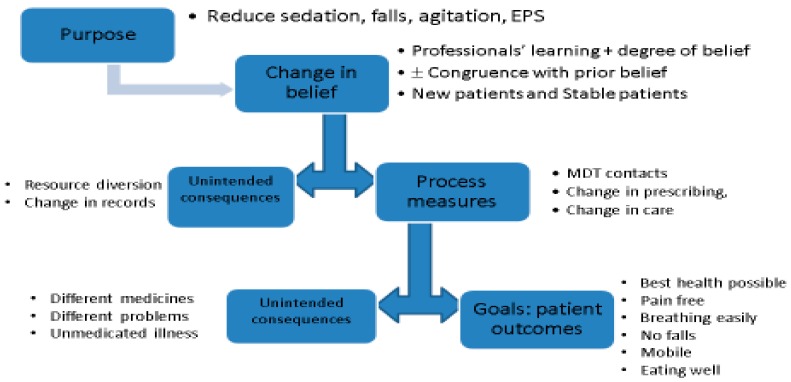
Logic Model for the introduction of regulation to reduce prescribing of antipsychotics to older adults. Real-world changes may follow a logic model of resources/actions/process change/outcome change (The Kellogg Foundation 1998). In any mandated intervention, there is an opportunity cost, and each phase may be associated with unintended consequences.

It remains to be seen how regulatory interventions will address the problems outlined and be placed in the public domain. However, there are no guarantees that regulatory interventions will improve the processes and outcomes of care, and structures are needed to pre-empt regulatory failure. Our logic model outlines the process of change—actions, beliefs, processes and outcomes—and possible unintended consequences (Figure 3).

### 5.1. Actions

While some regulatory actions are as straightforward as product withdrawal, many restrictions are complex, and therefore more difficult to target and monitor. For example, while prescribers are advised to restrict doses of paracetamol for people weighing <60 kg, and restrict further for those <50 kg [90], medicines are often prescribed without knowing patients’ current weight. Unless information on weight is passed to the prescriber, using ADRe or similar documentation, ascertaining such detail may be time-consuming, which results either in the restriction being overlooked or in the product falling out of favour. Many cautions and contraindications apply to subgroups; however, patients move between categories by aging, developing cardiovascular risk factors, or becoming pregnant. There is little indication that pre-emptive surveillance is widely undertaken, particularly in preconception care [91]. If de-prescribing is incongruent with prior beliefs of clinician or patient, change may be resisted and difficult [92]. Where regular monitoring is mandated, products may fall out of favour if the monitoring is thought to be too onerous (see ‘switching’, below). 

### 5.2. Change in Belief

Patients’ attitudes and commitments to their therapeutic regimen may influence prescribing: For example, a time series analysis found that admonitions had no impact on prescribing of opioids, intimating that patient preference or dependence may be influential [93]. Prescribers’ degree of belief in new evidence or interventions will determine transfer of evidence to individual patients, as is recognised by advocates of evidence-based medicine: research evidence can inform, but can never replace individual clinical expertise, and it is this expertise that decides whether the external evidence applies to the individual patient, and if so, how it should be integrated into a decision ([94], p. 72). The risks of unmedicated illness may override the risks of ADRs: for example, the prescription of antiepileptics was unaffected by warnings of suicidality [95]. Consultant psychiatrists responsible for clients with histories of severe mental illness, sometimes including homicides, are reluctant to change therapeutic regimens, despite disabling ADRs [84]. Systematic completion of a standard instrument like the ADRe Profile offers prescribers a record of all drug-related harms and symptoms of mental illness, such as irritability and hallucinations, and physical illness, such as chest pain and dyspnoea, to assist decision-making.

#### Learning and Unintended Learning

While regulation is directed at prescribing, there may be unintended impacts on other behaviours. Warnings of the impact of leukotriene receptor antagonists on mood and behaviour (aggression/hostility) increased prescriber and patient awareness, resulting in a rise in mental health contacts from 8.32 to 8.93%/month in adults, and suicide attempts from 0.06 to 0.09% in those aged 18–29 [96]. Spontaneous reporting overlooks ~95% of ADRs [97], leaving the data vulnerable to bias. Regulatory warnings increase spontaneous reporting, intensifying respondent and notoriety biases. For example, osteonecrosis of the jaw in 81 patients co-exposed to bisphosphonates and chemotherapy and/or corticosteroids was attributed solely to bisphosphonates [98]. In the French pharmacovigilance database, reports of adverse events rose in the 2 years following warnings; for example, reports of rhabdomyolysis more than doubled after this issue was highlighted in 2001 [99]. Increases in spontaneous reporting may reflect healthcare professionals’ learning, rather than real increases in ADRs [98,99]. ADRe depends on regular, routine monitoring, rather than spontaneous reports. 

### 5.3. Processes of Care

Prescribers’ and service users’ degree of belief, learning, and commitment to therapeutic intervention influence process measures, such as changes in prescribing, checking patients for restrictions, and engaging in close monitoring. Changes are contextualised by prior beliefs, entrenched attitudes, and perceptions of what is considered feasible and achievable [100]. 

#### Unintended Processes

Both service users and professionals may ‘work round’ restrictions or mandated monitoring. Where medicines are restricted or restrictions are seen to be inconvenient, patients may turn to buying over the internet. Alternative remedies may be sought from herbalists, suppliers of other complementary medicines, or even suppliers of recreational drugs. Anecdotally, where prescribers refuse to supply procyclidine to users of antipsychotic medicines, service users purchase this from street dealers or resort to feigning the signs of dystonia to prompt nurses to administer procyclidine under PRN (*pro re nata* or as needed) prescriptions. The UK’s Quality Outcomes Framework (QOF) aimed to link general practitioners’ (GPs) pay to quality of care [101]. This was expanded in 2006 to include completion of a questionnaire assessment of symptoms of depression, aimed at targeting prescribing to those more severely depressed [102]. However, this requirement for additional documentation was circumvented by using symptom rather than diagnostic codes [103]. 

Regulation may promote therapeutic substitution or switching. Additional regulatory requirements may encourage switching to (more expensive) medicines where patient monitoring is not mandated. For example, the prescription of factor Xa inhibitors increased between 2010 and 2014 [104], due, in part, to the reduced need to attend anticoagulation clinics [105]. Substitution of diclofenac for coxibs increased the use of diclofenac, until the recognition of the dose–response association of this medicine with cardiovascular events [106], and subsequent withdrawal from pharmacy sale [107].

Switching may not be problem-free. In the USA, guidelines to reduce prescribing of antipsychotics for older adults resulted in increased prescribing of benzodiazepines (adjusted Odds Ratio (aOR) 3.05, 1.17–7.94) and anti-dementia medicines (aOR 1.98, 1.12–3.50), but little change in use of antipsychotics (aOR 0.83, 0.37–1.89) (n = 331) [108]. ADRe aims to be comprehensive, capturing the undesirable effects of sedatives and other medicines, so it continues to be useful after therapeutic substitution to assess change or the emergence of different adverse effects.

### 5.4. Outcomes of Care

Changes to the processes of care do not necessarily improve outcomes. Outcomes, particularly mortality, are heavily confounded by lifestyle and genetic factors. Implementation of the UK’s QOF, associated with large monetary incentives, did not significantly improve mortality [109].

More comprehensive data are needed to capture all consequences of prescribing and regulatory change to minimise risk. Approximately 50% of mundane problems, such as constipation, xerostomia, dizziness, and incontinence, which may be drug-related, remain incompletely documented, reducing the likelihood of identifying both harms and benefits, unintended and intended, in practice and in pharmacovigilance database work [110]. Most ADRs are not medically serious, but these mundane problems, from incontinence to xerostomia, negatively impact quality of life or adherence to therapeutic regimens. Such problems are not medically interesting (an inverse interest law), and are only captured by proper surveillance. Direct questioning by professionals doubles the number of adverse drug events identified [111]. Primary care pharmacist questioning identified problems in 168/180 patients, for example, xerostomia, sweating, diarrhoea, constipation [112]. ADR reporting in clinical trials using standard questions and diary cards increased ADR reporting, for example, from 1.5–5.1% to 29–49% [113]. In USA primary care, 18% (394/2248) of patients reported ADRs to researchers, but only 3% (64) had previously had these documented [114]; incontinence and headache are among problems least likely to be documented [115]. When considered together, the diverse pharmacist-led medication therapy management programmes improve medication appropriateness, adherence, and dosages, but there is little evidence for their effectiveness in identifying and resolving drug therapy problems [71]. Nurse-led direct questioning using ADRe increased the mean number of problems recorded from 7.30 [SD 3.18] per participant to 15.81 [SD 5.90], and outcomes such as pain and falls improved [27]. The ADRe Profile addressed serious ADRs in ~10% patients in mental health teams [81,84], and in care homes [27,82].

#### Unintended Outcomes

The diagnostic uncertainty and social contingency inherent in recognition of unintended consequences suggest the need for more comprehensive, targeted data collection. Uncertainties surrounding complex restrictions are compounded by social and geographical distance between prescriber and patient, patients’ and professionals’ expectations (the Rosenthal effect), the mundane nature of many ADRs, and confounding by indication, co-prescription, or socioeconomic status. These impact on patients’ opportunities to report problems, the number and nature of problems recorded, and availability of data. Recognition is particularly difficult where outcomes are transgenerational, beyond the immediate neonatal period (education), or in vulnerable subgroups (e.g., co-exposed, polymedicated, immunosuppressed, or genetically vulnerable). 

Where medicines are changed or discontinued, unmedicated illness may emerge. The concerns regarding valproate increased prescribing of lamotrigine in pregnancy. However, enzyme induction in pregnancy demands dose adjustments; where these were not made, nine maternal deaths resulted [116]. A cluster randomised controlled trial (RCT) involving people with dementia in care homes (n = 177) found that a pharmacist review of antipsychotic medication reduced antipsychotic prescribing and led to a non-significant reduction in mortality (28% vs. 35%), but a worsening of neuropsychiatric symptoms [117] and quality of life measures, particularly negative emotions [118]. However, this unintended consequence of guideline compliance might be confined to the most disturbed, and withdrawal reduces the risk of falls [119]. By capturing all possible problems at regular intervals, ADRe promotes early recognition and reporting and improves our understanding of impact and outcomes.

One unintended outcome of regulatory interventions is to accentuate health inequalities.

Prescription of antipsychotics [74], selective serotonin re-uptake inhibitors (SSRIs) in pregnancy [120], and polypharmacy (>9 medicines) are concentrated amongst more deprived socioeconomic groups [121], leading to concerns that ADRs are contributing to health inequalities [122]. UK primary care is characterised by the Inverse Care Law [123], whereby resources are diverted away from areas of social deprivation and the greatest medical need. GPs in the most deprived areas are responsible for more patients and are under greater pressures and risk of burnout [124]. Working under such conditions may prove a barrier to implementation of regulations or warnings: Checks or new restrictions for medicinal products may be overlooked due to the urgency of care. The resources to monitor patients during withdrawal of medication may simply not be there, allowing outdated and suboptimal practices to persist. Therefore, to ensure that the most disadvantaged patients benefit, interventions need to be mandated and inspected. Accordingly, the Welsh Government has asked the Care Homes’ Inspectorate to check quarterly medication reviews of antipsychotic prescribing in care homes [62]. Were ADRe to be routinely utilised in this task, it would bring rigour and substance to these reviews. Its person-centred documentation of signs and symptoms will prevent this regulatory intervention being reduced to an audit of medicines’ charts. 

## 6. Conclusions

The reluctance to de-prescribe is based, in part, on the potential risks of medication withdrawal, and the associated commitment of resources, but perpetuates the ‘othering’ [18] and control of service users. For some medicines, ADRs are overtly stigmatising and disempowering—such as the agitation, nervousness, and anxiety caused by some antidepressants, and the movement disorders and cognitive impairment caused by antipsychotics. It is incumbent on healthcare professionals to ‘do no harm’, but the over-reliance on prescriptions can lead to real harm and an imbalance of power. Regulation at system level alone is not enough: Oversight should include comprehensive patient checks for undesirable adverse effects of medicines, however mundane. The ADRe Profile provides a sensible and usable tool to achieve improved patient care and increased understanding of the impact of ADRs.

## Figures and Tables

**Table 1:** Studies on the ADRe Profiles.

	Design	Clinical Area	Findings	Case Reports, Examples of ADRs Addressed without Hospitalisation
*Studies Using the ADRe Profiles*				
Jones et al. 2016 [81]	‘Before-and-after’ study of 20 patients	Community mental health, crisis resolution home treatment	The Profile identified previously unreported physical health problems for all participants, including two previously unreported potentially life-threatening problems (cardiac arrhythmia, and valproate-induced pancreatitis). In all, 4 participants had medicines discontinued, 3 were referred to consultant psychiatrists, 3 to general practitioners, 1 to ECG technicians, and 1 to dentists. Previously neglected health promotion issues were also recognised.	A middle-aged man, diagnosed with schizophrenia, had previously unrecorded but potentially serious cardiovascular problems (cardiac arrhythmia, intermittent acute chest pain) that worsened with exertion and radiated. He was referred immediately to his GP. The consultant determined that this case, and one other, fulfilled the criteria for a serious ADR, as it would have resulted in hospitalisation if unattended.
Jordan et al. 2015 [27]	Stepped wedge randomised controlled trial (RCT) over 7 months, 5 homes, 41 participants, 125 record reviews before Profile implementation and 124 after	Care home residents with permanent cognitive impairment	Profile administration increased the number of problems addressed from a mean of 6.02 [SD 2.92] to 9.86 [4.48], effect size 3.84, 95% CI 2.57–4.11, *p* < 0.001. For example, pain was more likely to be treated (adjusted Odds Ratio (aOR) 3.84, 1.78–8.30), and more patients attended dentists and opticians (aOR 52.76 (11.80–235.90) and 5.12 (1.45–18.03), respectively). Profile use was associated with reduction in mental health medicines (aOR 4.45, 1.15–17.22).	A lady in her late 80s, diagnosed with dementia at first administration of the Profile, was noted to be aggressive, restless, confused, sedated, and agitated. The ADRe Profile helped staff identify that hyoscine might be the cause. Hyoscine was discontinued. By the end of the study, 5 months later, aggression, restlessness, and sedation were no longer problems.
Jordan et al. 2014 [82]	Feasibility study, 11 patients’ records reviewed 3 times (before Profile implementation, after, and 3 months later), 3 homes. Feedback from clinicians	Care home residents with permanent cognitive impairment	The Profile took 20–25 min to implement, caused no harm, and supplemented usual care. On first use, the Profile identified previously undocumented problems for all service users: mean 12.7 [SD 4.7]. One month later, a mean of 4.9 [3.6] problems had been ameliorated. Clinical gains documented included: new prescriptions to manage pain (2 participants), psoriasis (1), Parkinsonian symptoms (1), rash (1); dose reduction of benzodiazepines for one service user; new care plans for oral hygiene, skin problems, and constipation.	A lady in her mid-60s, diagnosed with ‘Korsakoff’s syndrome’ and psoriasis, was noted to be oversedated. Benzodiazepine and antipsychotic prescribing were reduced, and sedation was no longer a problem at follow up. Itching rashes were also identified, more creams were administered, including an ‘as needed’ prescription for hydrocortisone, and symptoms were ameliorated.
Gabe et al. 2014 [80]	Parallel group RCT Researcher observed clinical visits before and after implementation of the Profile in the intervention arm. Feedback from patients and clinicians	Respiratory medicine, outpatient department, 54 patients recruited and followed up	The increase in numbers of problems per participant identified at follow up was significantly higher in the intervention arm where the median change was +20.5 (inter-quartile range (IQR) 13–26) while that in the control arm was −1 (−3 to +2) (Mann–Whitney U test: z = 6.28, *p* < 0.001). The increase in numbers of actions per participant taken at follow up was significantly higher in the intervention arm, where the median increase was +2.5 (1–4), while that in the control arm was 0 (−1.75 to +1) (Mann–Whitney U test: z = 4.40, *p* < 0.001).	Without the Profile, no actions were taken by nurses for a lady in her 50s, with respiratory problems sufficiently severe as to warrant oral prednisolone. Using the Profile, nurses advised her to contact her GP to seek advice regarding mood swings, depression, headaches, and immunisations. The nurse commented: “I would not have picked up on x’s depression without the Profile”.
Gabe & Jordan 2014 [83]	Inter-rater reliability Profiles completed in the presence of an observer	Respiratory medicine, outpatient department, 48 patients prescribed respiratory medicines	Cohen’s κ for inter-rater reliability for each item ranged 0.73–1 (good to complete agreement). The Profile identified previously unsuspected problems in all participants, including muscular weakness, skin, and mouth problems.	A lady in her 70s prescribed corticosteroids, bronchodilators, and other respiratory medicines, reported multiple oral problems, plus losing two stone in weight over the last six months, because her mouth was too sore to eat comfortably. She was advised to rinse her mouth shortly after each inhaler use, seek advice from the nurse for information on inhaler technique, and maintain routine dental check-ups.
Jordan et al. 2004 [76]	Comparison of instruments available to monitor antipsychotic medicines. Inter-rater reliability, 20 Profiles completed in the presence of an observer	Community mental health teams, 20 service users prescribed long-term medicines	The ADRe Profile assessed a broader range of physiological parameters and potential problems than other instruments. It is the only instrument with supporting information to prompt action in routine care. Items on the Profile had moderate-to-complete inter-rater reliability (ranging 0.44–1.00)	NA
Jordan et al. 2002 [84]	‘Before-and-after’ study with 1 intervention and 1 comparator group	3 community mental health teams in post-industrial South Wales, 40 service users prescribed long-term mental health medicines	Amongst the 20 clients in the intervention group, the Profile highlighted several problems, two of which were urgent. In the intervention group, the mean number of problems actioned per client increased from 0.35 (range = 0–4) without the Profile to 3 (range = 0–6) with (z = −3.747, 2 tailed *p* < 0.001). Nurses offered appropriate advice or encouraged clients to contact the relevant agencies to resolve the physical health problems identified. In the comparator group, the number of problems actioned declined from 0.85 (0–3) to 0.5 (0–2), a statistically insignificant difference (z = –1.47, *p* = 0.14).	Of 20 clients in the intervention group: One had coupled beats, and was urgently referred to the prescriber, who immediately reduced the dose of the antipsychotic depot. One had severe hypertension, 200/120 mmHg, and was immediately referred to his general practitioner (GP), and subsequently to renal physicians. Two had postural hypotension. They were encouraged to maintain adequate fluid intake. Notes were attached to the medical notes to alert the psychiatrist. Six had a degree of hypertension, above 140/90 mmHg. Measurements were repeated at three subsequent clinic visits. Five clients were advised to contact their GPs, one refused. The 6th client was being investigated for a cerebral tumour. Inflation of the cuff revealed marks of intravenous injections on the forearm of one client. There were no previous records of substance misuse..
Jordan 2002 [85]	‘Before-and-after’ study with intervention and comparator groups, 40 patients. Interviews with professionals and service users	Community mental health teams in post-industrial South Wales	Profiles apportioned aspects of medication management between nurses and medical prescribers. Most actions taken by nurses to alleviate adverse effects concerned clients’ physical health and advice on health-promotion. Nurses’ interventions would have been more effective had they been able to supply clients with certain medicines, for example for sunblock or oral care. For some clients, ameliorating the adverse effects of medication would have involved changes to prescribed antipsychotic medication; here, decisions were more equivocal.	One client was referred to his GP with chest pain; since he was receiving 100mg fluphenazine decanoate per week, the absence of an ECG recording contravened current guidelines. Nurse: You can attach this to the notes. Show the psychiatrist a copy. If you took the time to take it to the psychiatrist—it could work. (...) By using this we’ll have more evidence to show that there are side effects and we’re concerned, to get medication reviewed. Outpatient appointments are very ‘in and out’ and things get missed.
*Studies undertaken before the Profile was introduced*				
Jordan et al. 2000 [86]	Stakeholder interviews and 3 service user focus groups	Mental health nursing: 7 service user representatives, 3 service user focus groups	Service users described serious shortfalls in professionals’ abilities to inform them of common adverse effects of medication; these problems were attributable to inadequate educational preparation.	User group representative: CPNs (Community Psychiatric Nurses) focus on the psychiatric illness, they don’t see the medical side, or want to become involved. It’s to do with their training. They wouldn’t help with the constipation or the sunburn for my daughter. This should be in their training.
Jordan et al. 1999 [87]	Interviews, observations, and questionnaires with 14 community mental health nurses.	Community mental health teams	Service users were experiencing ADRs, but nurses did not have a structure to record and report problems. Doctors were seeking information from nurses, rather than directly from service users.	Nurse: There should be a form of structure for it (client education). It’s down to individuals whether or not they see the importance of educating people regarding their medication, and I think that should be part and parcel of the assessment. I think it should be there, and I know that it’s not, from my own experience. To me, whoever is on medication, I will ask them if they understand their medication. People say “Oh well, that’s the GP’s role, that’s the doctor’s role”, but it isn’t. It isn’t done and I always ask them that question, “Do you understand what your medication’s doing?”, and I suppose my knowledge maybe isn’t enough either, and I think that maybe I need more training to carry that further. (...) We’ve got to be prepared to answer questions—informed answers have got to be given, then people will ask, ‘What’s this for, what’s this supposed to do to me?’ (...)

ADRe was formerly known as the West Wales ADR Profile.

## Supplementary Materials

The following are available online at http://www.mdpi.com/2226-4787/6/3/102/s1, Table S1: Glossary.
