# Supplementary material for: Adverse Drug Reactions, Power, Harm Reduction, Regulation and the ADRe Profiles
Source: Pharmacy (Basel). 2018 Sep 18;6(3):102. doi: 10.3390/pharmacy6030102 (PMC6165166; doi:10.3390/pharmacy6030102)
Supplement: Supplementary file 1 [file pharmacy-06-00102-s001.pdf]

Supplementary material:

Table S1. Glossary.

Keyword	Definition	Reference
**ADE (adverse drug event)**	A portmanteau term encompassing ADRs, subtherapeutic effects of therapy, drug dependence, intoxications and untreated indications.<br>Based on: An injury resulting from medical intervention related to a drug.	Gyllensten et al. 2013 p. 2<br>Gyllensten H, Rehnberg C, Jönsson AK, Petzold M, Carlsten A, Andersson Sundell K. Cost of illness of patient-reported adverse drug events: a population-based cross-sectional survey. BMJ Open. 2013 Jun 20;3(6). pii: e002574. doi:10.1136/bmjopen-2013-002574. PubMed PMID: 23794552;<br>Bates DW, Cullen DJ, Laird N, et al. Incidence of adverse drug events and potential adverse drug events: implications for prevention. ADE Prevention Study Group. JAMA 1995; 274(1): 29–34
**ADR (adverse drug reaction)**	An adverse drug reaction (ADR) is any untoward and unintended response in a patient or investigational subject to a medicinal product * which is related to any dose administered.	[ICH], 1996) (Glossary)<br>International Conference on Harmonisation (ICH). *ICH Harmonised Tripartite Guideline for Good Clinical Practice*. Institute of Clinical Research, Marlow, Buckinghamshire. 1996. Available at: http://www.ich.org/fileadmin/Public_Web_Site/ICH_Products/Guidelines/Efficacy/E6/E6_R1_Guideline.pdf (Accessed January 31, 2018).
**AE (Adverse Event)**	Any untoward medical occurrence in a patient or clinical investigation subject administered a pharmaceutical product and which does not necessarily have a causal relationship with this treatment. An adverse event (AE) can therefore be any unfavourable and unintended sign (including an abnormal laboratory finding), symptom, or disease temporally associated with the use of a medicinal (investigational) product, whether or not related to the medicinal product.	[ICH], 1996)<br>International Conference on Harmonisation (ICH). *ICH Harmonised Tripartite Guideline for Good Clinical Practice*. Institute of Clinical Research, Marlow, Buckinghamshire. 1996. Available at: http://www.ich.org/fileadmin/Public_Web_Site/ICH_Products/Guidelines/Efficacy/E6/E6_R1_Guideline.pdf (Accessed January 31, 2018).
**Care home**	The OED defines 'care home' as a small institution providing residential accommodation with health or social services for the elderly, vulnerable children, the infirm, etc.<br>In the UK, care homes are considered as nursing or residential homes or mixed. Some specialise, for example, in care of the elderly mentally ill.	OED is available in hard copy and online<br>https://en.oxforddictionaries.com/definition/care_home<br>Further information can be found on the Regulations website<br>http://www.legislation.gov.uk/wsi/2002/324/contents/made

***Medicinal product**	Medicinal product is the portmanteau term used in the European Union defined as: *any substance or combination of substances presented as having properties for treating or preventing disease in human beings.*	(Directive 2001/83/EC3, Article 1(2)), Directive 2001/83/EC of the European Parliament and of the Council of 6 November 2001 on the Community code relating to medicinal products for human use. 2001. Available from: http://eur-lex.europa.eu/legal-content/EN/TXT/?qid=1420628195362&uri=CELEX:02001L0083-20121116 (Accessed March 13 2016).
**Preventable**	The OED defines 'prevent' as: anticipate or act in advance.<br>A wide variety of definitions, criteria, and instruments assess preventability of ADRs (Aronson 2012) and ADEs (Hakkarainen et al. 2013). Here, we report only the authors' assessments.	Hakkarainen KM, Andersson Sundell K, Petzold M, et al. Prevalence and perceived preventability of self-reported adverse drug events-a population-based survey of 7099 adults. PLoS One 2013; 8: e73166. doi: 10.1371/journal.pone.0073166 [published Online First: 2013/09/12] Reference 11<br>Aronson JK. Adverse drug reactions: history, terminology, classification, causality, frequency, preventability. Pp. 1–119 In: Talbot J., Aronson JK (eds) Stephens' Detection and Evaluation of Adverse Drug Reactions: Principles and Practice. Wiley-Blackwell, 6th edition, Chichester. 2012.
**Serious ADR**	Serious ADRs are those which result in death, life-threatening conditions, persistent or significant disability or incapacity, hospitalization, prolonged hospitalization, congenital anomalies (International Conference on Harmonisation 1996)	[ICH], 1996)<br>International Conference on Harmonisation (ICH). *ICH Harmonised Tripartite Guideline for Good Clinical Practice*. Institute of Clinical Research, Marlow, Buckinghamshire. 1996. Available online: http://www.ich.org/fileadmin/Public_Web_Site/ICH_Products/Guidelines/Efficacy/E6/E6_R1_Guideline.pdf (Accessed 2 January 2016).
**Side effects**	Dose-related, therapeutically-unrelated ADRs. The term is used without definition in the British National Formularly (BNF). The equivalent term in the SmPCs is 'undesirable effects'.	Edwards I.R., Aronson J.K. (2000) Adverse drug reactions: definitions, diagnosis, and management. *Lancet*: 356: 1255-9 P. 1255
